# Institutional Questions

**Published:** 1899-09-09

**Authors:** 


### INSTITUTIONAL QUESTIONS.

**An Invalid Bed.**

"INVENTOR" writes: Will you advise me as to the steps to take to obtain a patent for a new idea for an invalid or hospital bed?

You should take good advice on this subject, and be very sure that the improvements you mention have not been already made use of. People throw away a great deal of money, too often, upon securing patents for inventions which prove after all of no market value. Inquire at the Patent Office, of which we believe the address to be 19, Southampton Buildings, W.C.

**Consumption of Alcohol.**

"HOUSEKEEPER" writes: Can you tell me at all what is the approximate sum spent upon alcohol per head in general hospitals?

On page 95 of "Cottage Hospitals" (Scientific Press) you will find a table showing the average consumption of alcohol per head at a given number of general metropolitan and other hospitals. It is an item that varies considerably at different institutions, as a comparison of the reports of various hospitals will show to any observer; but in the table mentioned the summary gives the average cost per head per patient at 18 London and 21 cottage hospitals at 2s. 8d. per head at the former and 2s. 9d. at the latter.

**Space per Bed.**

"A CORRESPONDENT" asks: I shall be glad if you will kindly answer the following questions. In estimating the floor, wall, and cubic space per bed for a hospital ward, containing cots as well as beds, should the beds and cots be added together and the entire wall, floor, and cubic space be divided by that number? For instance, in a certain hospital one ward contains twelve beds, two cots, and a couch, making fifteen. Should the total wall space, floor space, &c., be divided by this number, and the result given as so many feet per bed?

This matter may be dealt with in two ways. 1. If a definite statement is to be made in a report, the total space must be divided by the total number of beds, cots, &c., and a note added pointing out that of the number given so many are cots for children under a certain age. 2. But if the information is needed as a guide to laying out a hospital, or arranging the beds in already existing wards, there are many things to be considered. In the Acts applying to lodging-houses, children under ten years of age are allowed only half the amount given to adults, an estimate which will not apply to hospitals, where the question is to a large extent one of floor and wall space. If it is a clinical hospital, it is just as important to have a clear space round a cot as round a bed; but even if not a clinical hospital, if it is thought proper in the case of adults that 8 feet or 9 feet shall intervene between the mouth of one patient and the mouth of the next, the same criterion should surely be applied to children. If, then, 1,500 cubic feet per patient be taken as the adult standard, the ward to be not more than 13 feet high, we cannot get our cubic feet without either having the wards more than 25 feet wide or the wall space more than 9 feet per patient. Usually the wards are made wider. In the newer general hospitals we think it will be found that the wall space allowed is about 8 feet or 9 feet per bed, and in hospitals for infectious diseases considerably greater than this. We have then to consider how much smaller a children's ward can be made without diminishing the wall space. Suppose we say that cots are 2 feet shorter than beds, and thus take our wards at 22 feet wide (which would be very narrow), instead of 26 feet, retaining the wall space at 9 feet as before. We then find that each cot in a children's ward so arranged would have 99 square feet of floor space, instead of 117, and 1,287 (11 by 9 by 13) cubic feet of space instead of 1,521 (13 by 9 by 13). Thus it will be seen that it is practically impossible to very materially reduce the cubic space for children without reducing the wall space. Indeed, when children are to be placed with adults it is quite impossible to do so at all, the height and width of the ward being already fixed. If we are to maintain that children should be kept apart, mouth from mouth, as far as adults, it is clear that we cannot, without unduly narrowing the ward or making it very low, allow a diminution of the air space of more than about one-sixth from the amount provided in the case of adults. In practice, however, a greater reduction has been allowed, and wall space reduced to about 7 feet per cot, and the cubic space to about 1,000; and we are not prepared to say that any injury has resulted. On the other hand, we must remember that in dealing with infectious disease, such as diphtheria, the space between the beds has been very largely increased, even in wards mainly occupied by children.

